# Pernicious Anæmia

**Published:** 1889-11-16

**Authors:** 


### PERNICIOUS ANÆMIA.

In the September number of the *Practitioner*, Dr. William Hunter, John Lucas Walker Student in Pathology at the University of Cambridge, makes some "observations on the urine in pernicious anæmia." The main feature of the case from which urine was obtained was gradual increasing weakness without any evident cause, and without any corresponding degree of emaciation. The patient had, however, recurrent sore throat, presenting patches of fiery redness. Gastric symptoms were absent throughout, but during the latter part of the illness there were attacks of diarrhœa, and towards the last the patient presented a well-marked lemon colour. Epistaxis also occurred, chiefly in the earlier stages of the malady, which altogether lasted about a year and a half. At the time Dr. Hunter saw the patient, June 1888, the red corpuscles numbered 64 per cent., with 56 per cent. of hæmoglobin, which latter high percentage was maintained throughout. This Dr. Hunter considers to be the only characteristic feature presented by the blood in pernicious anæmia, as it is not met with in anæmia from loss of blood or in chlorosis. After death the spleen was found enlarged, the liver fatty and mottled, but no trace of malignant disease in any organ. The urine was acid throughout the case, with a S. G. of 1,010 to 1,020. The quantity passed daily varied from 40 to 52 ounces; the colour was always exceedingly high, but the fluid was perfectly clear and transparent. This colour of the urine could not be traced to the pigments. By pectroscopic examination and by other means, the colouring matter in the urine was diagnosed as *pathological urobilin.* The presence of this colouring matter Dr. Hunter states has a twofold significance. *Pathological urobilin* is derived from the disintegration of hæmoglobin, from which the conclusion is drawn that pernicious anæmia depends on excessive destruction of blood; in other words, that pernicious anæmia is hæmolytic in character. Now various observers have regarded pernicious anæmia as in some obscure manner a different malady to ordinary anæmia. But, in our opinion, this so termed pernicious anæmia should only be regarded as an anæmia in which the symptoms are more intense, and a fatal termination more probable. The intense form of anæmia has, in our experience, afforded an aggravation of the apparent causes, and changes met with in the less intense varieties of the malady. It is true that in ordinary anæmia the urine is more often pale than not from diminution of pigment; and until further investigation is made it may, perhaps, be permissible to regard the high coloured urine observed in this one case by Dr. Hunter as an accidental coincidence.

